# Impact of tobacco smoking and alcohol consumption on the treatment efficacy among psoriasis patients: A study protocol

**DOI:** 10.18332/tid/195380

**Published:** 2024-11-20

**Authors:** Fanlingzi Shen, Yuning Ding, Ruiping Wang

**Affiliations:** 1 Clinical Research Center, Shanghai Skin Diseases Hospital, School of Medicine, Tongji University, Shanghai, China; 2 School of Public Health, Shanghai University of Traditional Chinese Medicine, Shanghai, China

**Keywords:** psoriasis, tobacco, alcohol, treatment response

## Abstract

Psoriasis is a common skin disease. With an in-depth understanding of psoriasis, small-molecule drugs and biologics are developed and used in clinical practice, but some patients still cannot achieve a satisfactory therapeutic effect. Tobacco smoking and alcohol drinking are proven to be factors affecting psoriasis. Still, evidence of the impact of tobacco smoking and alcohol consumption on the efficacy of psoriasis treatment is limited. This study aims to understand the prevalence of tobacco smoking and alcohol drinking among patients with psoriasis in Shanghai and to examine the association between tobacco smoking as well as alcohol drinking and the therapeutic effect in patients with psoriasis. We conducted a longitudinal observational study and recruited at least 500 psoriasis patients at Shanghai Skin Disease Hospital. In this study, patients with clinically diagnosed psoriasis vulgaris, aged ≥18 years, both males and females, with informed consent were recruited. However, patients with pregnancy, serious underlying disease conditions, communication barriers, and violation of medication regulations were excluded. Patients with psoriasis in this study receive a physical examination and case record form interview. The primary outcome indicator is the proportion of patients with PASI_75_ achievement at Week 8. In this study, we use SAS 9.2 software to analyze the data. This study has been reviewed and approved by the Institutional Ethics Review Committee of Shanghai Skin Disease Hospital in 2021 (NO. 2021-44). It has been registered in the Chinese Clinical Trial Registry (ChiCTR2200066403). Patient recruitment began in January 2021 and is proposed to be finished in December 2024. The findings in this study will provide evidence of how tobacco smoking and alcohol drinking impact the treatment efficacy among patients with psoriasis. Therefore, the implementation of tobacco control and alcohol abstinence benefit the improvement of treatment responses.

## INTRODUCTION

Psoriasis is a complex chronic recurrent inflammatory skin disease that seriously endangers human health^1,2^. Psoriasis is characterized by infiltration of inflammatory cells, abnormal proliferation and differentiation of epidermal keratinocytes, and capillary dilatation^3^. The occurrence and development of psoriasis^4^ is a combined action of many factors, including genetics, immunity, and environmental factors, etc. Previous studies indicate that the prevalence of psoriasis ranges from 0.14% to 1.99%, with a lower prevalence in Asia (0.14%)^5^. A study shows that the standardized prevalence of psoriasis in China was 0.47% in 2012^6^. In recent years, due to the increased prevalence, long disease duration, and serious disease burden, psoriasis is treated as an important public health issue.

In the past century, significant progress has been achieved in the pathogenesis of psoriasis. The activation, migration, and release of related cytokines of T cells play a key role in forming psoriasis lesions^1,7,8^. With continuous deepening research on the immune mechanism of psoriasis, small-molecule drugs, and biologics have been developed and applied to clinical practice. Clinical evidence has shown that small-molecule drugs and biologics can alleviate psoriasis symptoms, providing patients with more effective treatment options^4,9^. Although biologics are gradually becoming the first-line systemic treatment choice for patients with moderate-to-severe psoriasis, the high treatment costs limit its widespread use in Chinese patients with psoriasis. So, methotrexate, acitretin, and narrow-band ultraviolet B (NB-UVB) are still the preferred therapies for patients with psoriasis in China.

Previous studies indicate that many factors play a role in the development of psoriasis, and growing evidence indicates that ultraviolet radiation, tobacco smoking, and alcohol drinking are vital triggers for psoriasis initiation^10,11^. Tobacco smoking and alcohol drinking are common adverse lifestyle behaviors and are also considered to be closely associated with psoriasis occurrence and disease progression^12^. Previous studies have shown an increased prevalence of psoriasis among smokers (OR=1.84; 95% CI: 1.4–2.3), and the risk of psoriasis is increased among those with higher daily smoking frequency, with more smoking years and smoking pack-years^13,14^. Moreover, alcohol drinking is also considered a trigger or an aggravating factor for psoriasis. A prospective study implemented in the United States found that alcohol drinking could increase the risk of psoriasis occurrence by 1.72 times^15^. Meanwhile, studies have also shown that alcohol consumption can lead to a reduced response to psoriasis treatment, which is detrimental to prognosis^11^.

China has a long history of wine culture, and studies have shown a dramatic increase in alcohol consumption and alcohol-related social and health problems in China^16^. Additionally, China is also the world’s largest producer and consumer of tobacco, with 300 million smokers and 1 million deaths annually due to tobacco consumption^17^. The high prevalence of tobacco smoking and alcohol consumption leads to a heavy disease burden in China. As aforementioned, tobacco smoking and alcohol drinking are common risk factors which are closely related to the initiation and development of psoriasis. However, there is still limited evidence on how tobacco smoking and alcohol drinking affect the treatment efficacy in patients with psoriasis, especially in China^8,18-20^.

In this study, we will conduct a longitudinal observational study to understand the prevalence of tobacco smoking and alcohol drinking among patients with psoriasis in Shanghai and to examine the association between tobacco smoking as well as alcohol drinking and the therapeutic effect in patients with psoriasis.

## METHODS

### Study setting

This longitudinal observational study is conducted and reported in accordance with the guidance for strengthening the reporting of observational studies in epidemiology^21^. The study was scheduled to initiate the recruitment of patients with psoriasis in January 2021 and is proposed to be finished in December 2024. All data are collected at Shanghai Skin Disease Hospital. This study has been reviewed and approved by the Institutional Ethics Review Committee of Shanghai Skin Disease Hospital in 2021 (NO.2021-44). It has been registered in the Chinese Clinical Trial Registry (ChiCTR2200066403). The implementation of this study strictly adheres to the Declaration of Helsinki.

### Study population

In this study, psoriasis vulgaris is diagnosed and confirmed according to the guidelines of the Chinese Clinical Dermatology, which is in line with the global guidelines for psoriasis diagnosis and treatment^22^. The patient inclusion criteria cover: 1) aged ≥18 years; 2) both male and female patients with psoriasis; and 3) moderate to severe psoriasis (body surface area, BSA ≥3, and psoriasis area and severity index, PASI ≥3). The patient exclusion criteria cover: 1) pregnant or lactating women; 2) patients with serious primary diseases, including cardiovascular and hematopoietic disorders, as well as psychiatric illnesses; 3) patients with communication barriers; 4) patients who fail to adhere to medication regulations and cannot assess treatment efficacy; and 5) patients who have incomplete data, which impedes the evaluation of treatment efficacy. All psoriasis patients are informed of the study objective, study procedure, and the benefits and potential risks of this study ahead of their informed consent signature.

### Sample size

This is a longitudinal observation study, and we use the sample size formula:

*n=[μ_α_^2^×p(1-p)]/δ^2^*

to calculate the sample size. Based on our pre-survey, we assume that the prevalence of tobacco smoking or alcohol drinking among patients with psoriasis is 30% in Shanghai, so we set p=30%, α=0.05, δ=15% of p, and a non-response rate of 10%; the sample size calculation indicates that at least 445 patients with psoriasis should be enrolled. So, we plan to enroll at least 500 patients with psoriasis in this study.

### Research procedure

In this study, all enrolled patients with psoriasis sign an informed consent form and are then examined and evaluated by a dermatologist at their first hospital visit, including PASI, BSA, and physician global assessment (PGA). After a physical examination and psoriasis disease evaluation, dermatologists make a treatment plan based on each patient’s condition. The treatment plan covers five options: acitretin treatment (25–50 mg daily), methotrexate treatment (15–20 mg per week, with folic acid supplementation), NB-UVB (2–4 times weekly), benvitimod treatment (2 times per day), and biologics treatment (ustekinumab, risankizumab, secukinumab, etc.).

After the treatment plan is made and assigned, each patient receives a follow-up at weeks 4 and 8. Moreover, dermatologists evaluate the patient’s condition (PASI, BSA, and PGA) at weeks 4 and 8, respectively. In addition, adverse events related to the treatment, such as itching, pain in the treated area, erythema, fatigue, and nausea, are examined and recorded during the eight weeks of follow-up.

### Data collection

This study collected data through a self-designed case report form (CRF) administrated by dermatologists with unified training. The CRF covers six parts: 1) demographic features, including age, sex, education level, marital status, monthly individual income, ethnicity, height, and weight; 2) non-communicable disease (NCD) comorbidity among psoriasis patients, including type 2 diabetes, hypertension, coronary heart disease, non-alcoholic fatty liver, etc.; 3) history of psoriasis, including the initiation age of psoriasis, disease duration, psoriasis recurrence season and familial aggregation; 4) lifestyle habits such as tobacco smoking, alcohol drinking, physical exercise, and sleep status, etc.; 5) psoriasis severity (BSA, PASI, and PGA) at week 0 as baseline, and at weeks 4 and 8 after treatment; and 6) dermatology life quality index (DLQI) and hospital anxiety and depression scale (HADS) at baseline (week 0).

### Outcome measurement

*Primary outcome*

In this study, the primary outcome indicator is set as the proportion of patients with the achievement of PASI_75_ at week 8. The PASI_75_ is defined as patients achieving ≥75% improvement in PASI score and is calculated by the formula: [(PASI at baseline - PASI at week t)/PASI at baseline] ×100%.

*Secondary outcome*

In this study, secondary outcome indicators include the prevalence of tobacco smoking, the prevalence of alcohol drinking, scores of PASI, BSA, PGA, DLQI, and HADS among patients with psoriasis. Detailed information for the calculation of secondary outcome indicators is as follows.

### Prevalence of tobacco smoking and alcohol drinking

In this study, we define a smoker as a person who smoked at least 100 cigarettes in his/her lifetime, a current smoker as someone who still smokes at the time of investigation, and a former smoker as someone who has stopped smoking for at least three months at the time of investigation. The prevalence of tobacco smoking is calculated as the number of smokers divided by the total number of patients with psoriasis. Likewise, we define an alcohol drinker as a person who has drunk alcohol at least twice a week for at least six months in his/her lifetime, a current alcohol drinker as someone who still drinks at the time of investigation, and a former drinker as someone who has stopped drinking for at least three months at the time of investigation. The prevalence of alcohol drinking is calculated as the number of drinkers divided by the total number of patients with psoriasis.

### PASI scores

PASI score is commonly used to quantitatively evaluate the condition of psoriasis, which is based on the severity of erythema (E), infiltration (I), desquamation (D) of the patient’s skin lesions, and the affected area of involvement of the skin lesions in the head and neck (H), upper limbs (U), trunk (T), and lower limbs (L). The PASI score is calculated as:

H×(E+I+D)×0.1 + U×(E+I+D)×0.2 + T×(E+I+D)×0.3 + L×(E+I+D)×0.4

in which the affected area of involvement of skin lesion (H, U, T, L) is evaluated through a seven-point Likert scale: 0 = 0%, 1 = <10%, 2 = 10–29%, 3 = 30–49%, 4 = 50–69%, 5 = 70–89% and 6 = 90–100%. The severity of erythema (E), infiltration (I), desquamation (D) is assessed through a five-point Likert scale: 0 = none, 1 = mild, 2 = moderate, 3 = severe, and 4 = extremely severe. The PASI score ranges 0 to 72, with a higher score suggesting that patients are more ill^23^. In this study, the PASI is assessed at baseline (week 0), week four, and week eight after treatment.

### BSA scores

BSA is usually estimated in terms of the palm of the hand, with one palm area of the patient being equivalent to 1% of the body surface area. BSA score is the combination of the affected area of involvement of skin lesions in the head and neck (H), upper limbs (U), trunk (T), and lower limbs (L). This study assesses the BSA score at baseline (week 0), week four, and week eight after treatment.

### PGA scores

PGA is a comprehensive assessment of the severity of patients with psoriasis based on erythema (E), infiltration (I), and scales (S). The severity of erythema (E), infiltration (I), and scales (S) are assessed through a six-point Likert scale: 0 = none, 1 = almost clear, 2 = mild, 3 = moderate, 4 = severe, and 5 = extremely severe, with higher scores indicating more severe illness. PGA is calculated^24^ as (E+I+S)/3. This indicator is assessed at baseline (week 0), week four, and week eight after treatment.

### DLQI scores

DLQI focuses on the various impacts of skin diseases on patients in the last week, which mainly covers six dimensions, including physiological response, psychological feeling, family, interpersonal communication, occupational restrictions, social activities, and treatment response. DLQI covers ten questions, and all questions are scored through applying a 4-point rating system (0, 1, 2, 3). DLQI score is obtained by summing up the scores of each question, which range 0 to 30, with a higher score suggesting that patients with lower quality of dermatology life^25^. In this study, the DLQI score is only assessed at baseline (week 0).

### HADS scores

The HADS consists of the anxiety subscale (HADS-A) and the depression subscale (HADS-D), both of which have seven items. HADS-A and HADS-D are assessed through a four-point Likert scale: 0 = none, 1 = mild, 2 = moderate, and 3 = severe. The score of HADS-A and HADS-D ranges 0 to 21, with higher scores suggesting that patients with more anxiety and depression, respectively^26^. This study assesses HADS-A and HADS-D at baseline (week 0).

### Participant timeline

**Figure 1:**
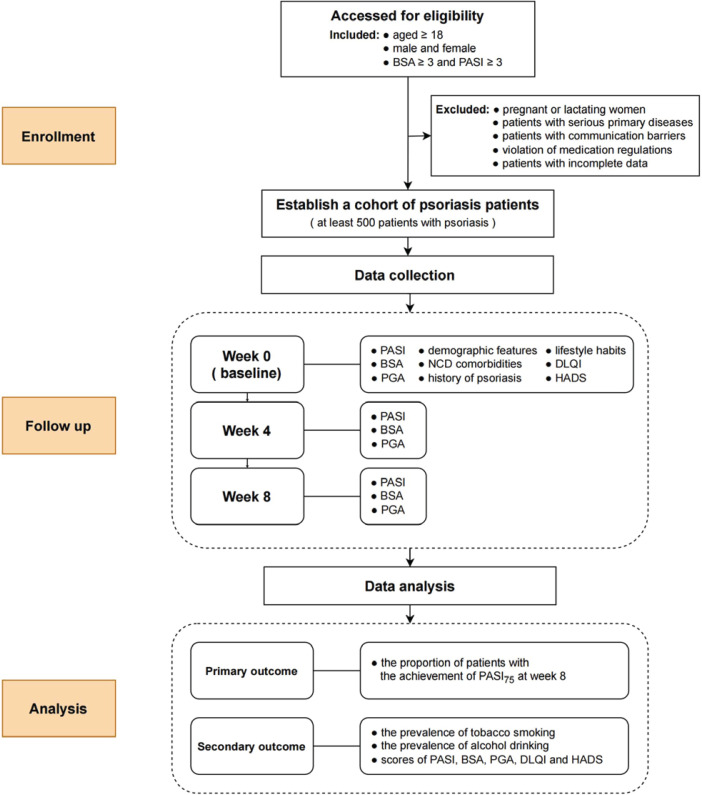
Flow diagram of the impact of tobacco smoking and alcohol drinking on the treatment efficacy among psoriasis patients at Shanghai Skin Disease Hospital from 2021 to 2024

In this study, psoriasis patient recruitment started in January 2021 and is proposed to be finished in December 2024 (Figure 1). The detailed information for data collection and the evaluation of the patient’s disease severity at baseline (week 0) and weeks four and eight after the treatment is provided in Table 1.

**Table 1:** Schedule of the enrollment and assessment of impact of tobacco smoking and alcohol consumption on the treatment efficacy among psoriasis patients at Shanghai Skin Disease Hospital from 2021 to 2024

*Schedule*	*Enrollment*	*Follow-up*	
	*Week 0*	*Week 4*	*Week 8*
**Enrollment**			
Eligibility screen	×		
Informed consent	×		
**Outcome assessments**			
PASI_75_ achievement			×
The prevalence of tobacco smoking	×		
The prevalence of alcohol drinking	×		
PASI scores	×	×	×
BSA scores	×	×	×
PGA scores	×	×	×
DLQI scores	×		
HADS scores	×		
**Safety assessments**			
AE		×	×

PASI_75_: at least 75% PASI reduction at week 8 compared with the PASI at baseline. PASI: Psoriasis Area and Severity Index. BSA: body surface area. PGA: Physician Global Assessment. DLQI: Dermatology Life Quality Index. HADS: Hospital Anxiety and Depression Scale. AE: adverse events.

### Quality control

In this study, we implement a series of measures to improve data quality and ensure the reliability of collected data. First, we implement a pilot study to train all investigators participating in this study and to evaluate the reliability and validity of the CRF used for data collection. Second, we assign two doctoral students as the inspectors to conduct comprehensive checks to ensure the accuracy and integrity of the collected data. Third, we actively contact all recruited patients during the 8 weeks of follow-up, as frequent communication will enhance patients’ trust and increase their willingness to participate and stay in the study.

### Statistical analysis

In this study, all data are recorded in the CRF and then input into a database established by EpiData software version 3.1 (EpiData Association, Odense, Denmark), and SAS software version 9.2 (SAS Institute Inc. Cary, NC, USA) is applied for data analysis. In this study, mean and standard deviation (SD), median, and interquartile range (IQR) are used to describe quantitative variables as appropriate, and frequencies (n) and proportions (%) are used to describe qualitative variables. We apply a two-sample Student’s t-test or Wilcoxon rank-sum test to examine the difference between groups for quantitative variables as appropriate, and a Wilcoxon rank-sum test, chi-squared test, or Fisher’s exact test is used to examine the difference between groups for qualitative variables. The multivariable logistic regression model is applied to explore the determinants influencing the treatment efficacy in patients with psoriasis. Odds ratios (OR) and 95% confidence intervals (95% CI) are calculated to assess the association between tobacco smoking, alcohol drinking, other pertinent factors, and the PASI_75_ achievement at week eight among patients with psoriasis. The linear mixed model assesses changes in PASI scores and interaction between time and group (type of treatment, smoking status, etc.). We conduct subgroup analysis based on treatment options and gender. In this study, we conduct univariate analysis and select variables as potential confounders if p<0.05. These selected confounders are adopted into the multivariable logistic regression model and the linear mixed model for adjustment. In this study, a p<0.05 (two-tailed) is considered statistically significant.

## DISCUSSION

Psoriasis vulgaris is a common disease that usually has systemic manifestations. Although advances in technology and research have greatly promoted the development and clinical application of new drugs that benefit more patients with psoriasis, there are still no absolute cure measures for psoriasis. The itching, redness, and scales caused by psoriasis lesions not only affect the appearance of the patients’ skin and cause social distress, but also affect their sleep and concentration and significantly impact their quality of life. Lifestyle factors, including tobacco smoking, alcohol drinking, high fatty diet, and lack of physical exercise, are not only closely related to human health but also play a role in the initiation and development of psoriasis. However, there is limited evidence on how lifestyle factors will affect the treatment efficacy in patients with psoriasis, especially in China. Therefore, actively exploring the impact of lifestyle exposure factors on the efficacy of psoriasis treatment is significant.

This protocol outlines the basic principle and design of a longitudinal observational study based on a cohort with an enrollment of ≥500 patients with psoriasis. We plan to explore the impact of lifestyle factors, including tobacco smoking and alcohol drinking, on the treatment efficacy among patients with psoriasis, to provide multifaceted intervention and effective treatment recommendations for them. To our knowledge, this is the first study with the recruitment of over 500 psoriasis patients to explore the relationship between tobacco smoking, alcohol drinking, and treatment efficacy among psoriasis patients based on clinical practice in China. In addition, the diagnosis, severity determination, and treatment plan of psoriasis will be conducted by dermatologists rather than the self-reports of patients, which ensures the reliability and accuracy of the study. Moreover, the clinical data of patients with psoriasis will be extracted directly from the Health Information System (HIS) and so will not involve recall bias, resulting in high data quality.

This study is expected to provide several key results. The findings include the prevalence of tobacco smoking and alcohol drinking among patients with psoriasis in Shanghai, the association between tobacco smoking as well as alcohol drinking, and the therapeutic effect at week 8 in patients with psoriasis. Based on these findings, we can make targeted recommendations for disease control and prevention among patients with psoriasis, which not only contribute to the personalized treatment for individual patients but also provide important information and guidance for health policy research, all of which is beneficial to the long-term diseases treatment and management among patients with psoriasis.

### Limitations

This study may have some limitations. First, patients with psoriasis will be recruited in one hospital in Shanghai, which ensures high internal validity but may limit the generalization of the findings to other areas and countries. Second, lifestyle factors such as tobacco smoking, alcohol consumption, high fatty diet, and lack of physical exercise are collected through face-to-face interviews, which may result in recall bias and report bias. Third, we do not conduct biochemical confirmation of patients’ smoking and drinking behavior (such as the concentration of cotinine in urine and phosphatidylethanol in the blood, respectively) but based on patients’ self-reports. This may induce information bias, thereby affecting the evaluation of the influence of tobacco smoking and alcohol drinking on the treatment efficacy to some degree. Fourth, in this study, we will conduct eight weeks of follow-up among psoriasis patients with systematic treatment (acitretin, methotrexate, NBUVB, benvitimod, and biologics), which will ensure 100% compliance. However, the evaluation of the treatment efficacy among psoriasis patients with biologics is usually set as 12 weeks or longer due to its treatment schedule, which may lead to the under estimation of the treatment efficacy of biologics and decrease the comparability of this study with other studies. Fifth, residual confounding factors may also influence the results. Sixth, the limitation of the study design and the lack of randomization may lead to selection bias, thereby affecting the inference of causal relationships.

## CONCLUSIONS

This longitudinal study investigates the prevalence of lifestyle exposure factors in psoriasis patients and explores their impact on the treatment efficacy. An 8-week follow-up is conducted, with the proportion of patients with the achievement of PASI_75_ at week eight as the main outcome measure to explore the impact of risk factors on the treatment efficacy. The results of this study may provide insights into the relationship between lifestyle exposure factors and treatment efficacy and provide a basis for personalized, comprehensive management of psoriasis, thereby optimizing patient treatment outcomes, improving quality of life, and reducing disease burden.

## Data Availability

The data supporting this research are available from the authors on reasonable request.
